# Melanoblast development coincides with the late emerging cells from the dorsal neural tube in turtle Trachemys scripta

**DOI:** 10.1038/s41598-017-12352-0

**Published:** 2017-09-21

**Authors:** Ritva Rice, Judith Cebra-Thomas, Maarja Haugas, Juha Partanen, David P. C. Rice, Scott F. Gilbert

**Affiliations:** 1 Developmental Biology, Institute of Biotechnology, University of Helsinki, Helsinki, Finland; 2 Orthodontics, Department of Oral and Maxillofacial Diseases, University of Helsinki, Helsinki, Finland; 3 Department of Biology, Millersville University, Millersville, PA USA; 4 Department of Genetics, University of Helsinki, Helsinki, Finland; 5 Orthodontics, Oral and Maxillofacial Diseases, Helsinki University Hospital, Helsinki, Finland; 6 Department of Biology, Swarthmore College, Swarthmore, PA USA; 7 Institute of Biomedicine and Translational Medicine, University of Tartu, Tartu, Estonia

## Abstract

Ectothermal reptiles have internal pigmentation, which is not seen in endothermal birds and mammals. Here we show that the development of the dorsal neural tube-derived melanoblasts in turtle *Trachemys scripta* is regulated by similar mechanisms as in other amniotes, but significantly later in development, during the second phase of turtle trunk neural crest emigration. The development of melanoblasts coincided with a morphological change in the dorsal neural tube between stages mature G15 and G16. The melanoblasts delaminated and gathered in the carapacial staging area above the neural tube at G16, and differentiated into pigment-forming melanocytes during *in vitro* culture. The Mitf-positive melanoblasts were not restricted to the dorsolateral pathway as in birds and mammals but were also present medially through the somites similarly to ectothermal anamniotes. This matched a lack of environmental barrier dorsal and lateral to neural tube and the somites that is normally formed by PNA-binding proteins that block entry to medial pathways. PNA-binding proteins may also participate in the patterning of the carapacial pigmentation as both the migratory neural crest cells and pigment localized only to PNA-free areas.

## Introduction

In cold-blooded reptiles, such as turtles, the pigmentation and its patterning in the integument facilitates cryptic coloration, thermoregulation, and social signaling^1^. All vertebrates share a single type of pigment cell: the neural crest-derived melanocyte that accumulates melanin^1,2^. The neural crest is a transient, multipotent and migratory cell population, and its conserved gene regulatory network evolved more than 500 million years ago in a common vertebrate ancestor^3,4^. The neural crest develops along the dorsal neural tube, and the location along the axis and the migratory pathway that the neural crest cells follow affect the types of derivatives arising from the multipotent neural crest cells^5,6^. Trunk neural crest generates, for instance, neuronal and glial cells of the peripheral nervous system and melanocytes. The medially migrating trunk neural crest cells (NCCs) become glial or neuronal cells. Trunk NCCs that become melanocytes are among the last neural crest cells to emerge from the trunk region, and they migrate along a dorsolateral route between the surface ectoderm and the somite^7–9^. Melanoblasts, the progenitor cells of melanocytes, arise from the pluripotent trunk NCCs that become gradually fate-restricted; the pluripotent trunk NCCs generate bipotent neural-glial and glial-melanogenic precursor cells. The fate-restricted bipotent glial-melanogenic precursor cells divide to make melanoblasts^8^. In ectothermal anamniotes (fish and amphibians) melanoblasts can travel along the medial pathway, and in ectothermal amniotes (reptiles) extracutaneous melanoblasts are found in locations that parallel the locations of medially migrated neural crest cells^8,10–12^.

The development and fate of the neural crest cell-derived melanoblasts results from a complex gene regulatory network that is highly conserved among vertebrates^3,8^. A forkhead transcription factor FoxD3 together with SoxE subgroup transcription factors Sox9 and Sox10 specify dorsal neuroepithelial cells as neural crest cells^13^. Epithelial-mesenchymal transition (EMT) leads to delamination of the neural crest cells, and this is driven for example by zinc-finger transcription factors, such as Snail2/Slug^13,14^. Early migratory cells still express transiently some EMT markers such as Slug, and later migratory cells are HNK-1 positive^9,15–19^. The fate of the neural crest cells depends on changes in the cells themselves and on the environmental cues along their route. For instance, FoxD3 expression in the early trunk neural crest cells together with environmental inhibitory signals, such as peanut agglutinin (PNA)-binding molecules, present in the dorsal surface ectoderm, prevents these cells from entering the dorsolateral pathway^20^. The repression of *FoxD3* expression and the continued expression of the neural crest specifier gene *Sox10* in the trunk neural crest cells allows the expression of a transcription factor *Mitf*, and melanoblast differentiation is switched on^8,21–23^. Delamination of the melanoblasts coincides with the expression of *Mitf* in the neural crest cells and a loss of the inhibitory PNA-binding molecules on the surface ectoderm, thus making it permissive for the melanocytic neural crest cells to enter the dorsolateral pathway^20^. Migratory *Mitf*-expressing melanoblasts accumulate in the migration staging area (MSA), or the carapacial staging area (CSA) in turtles^24^, between the neural tube and the somites prior to entering the pathway^25,26^.

In reptiles, melanocytes are found not only in skin, as in birds and mammals, but also in extracutaneous tissues^10^. In the soft-shelled turtle *Trionyx sinensis japonica*, migratory HNK-1 positive neural crest cells have been shown to migrate medially between the anterior portion of the dermomyotome and the sclerotome at developmental stage 17 to 18. At stage 11, trunk neural crest cells also migrate between the surface ectoderm and the differentiated somite. However, while these NCCs can be detected in the early wave of turtle trunk NCC migration, DOPA (L-3, 4-dihydroxyphenylalanine)-reactive melanoblasts are first seen in the dermis and myotome, and above the neural tube at developmental stage 17^10^, that is, during the second wave of trunk NCC emigration from the neural tube^24,27^. Stage 9 or 10 isolated trunk neural crest cells from the soft-shelled turtle lacked melanocyte differentiation during cell culture unless the cells were cultured in high concentration of serum and embryo extract or by addition of melanocyte stimulating hormone^28^. We have previously shown that in the hard-shelled turtle *Trachemys scripta*, HNK-1 positive late migratory trunk neural crest cells are visible above the neural tube from stage G16 through to G19 within the loosely packed carapacial mesenchyme above the dorsal neural tube^24,27^. As the DOPA-positive melanoblasts were visible on and above the neural tube at stage 17 in the soft-shelled turtle^10^, we became interested in whether the late emigrating trunk neural crest cells include precursors to melanocytes in turtles.

## Results

### Morphology of the neural tube and emigration of late trunk neural crest cells in *Trachemys scripta*

**Figure 1:**
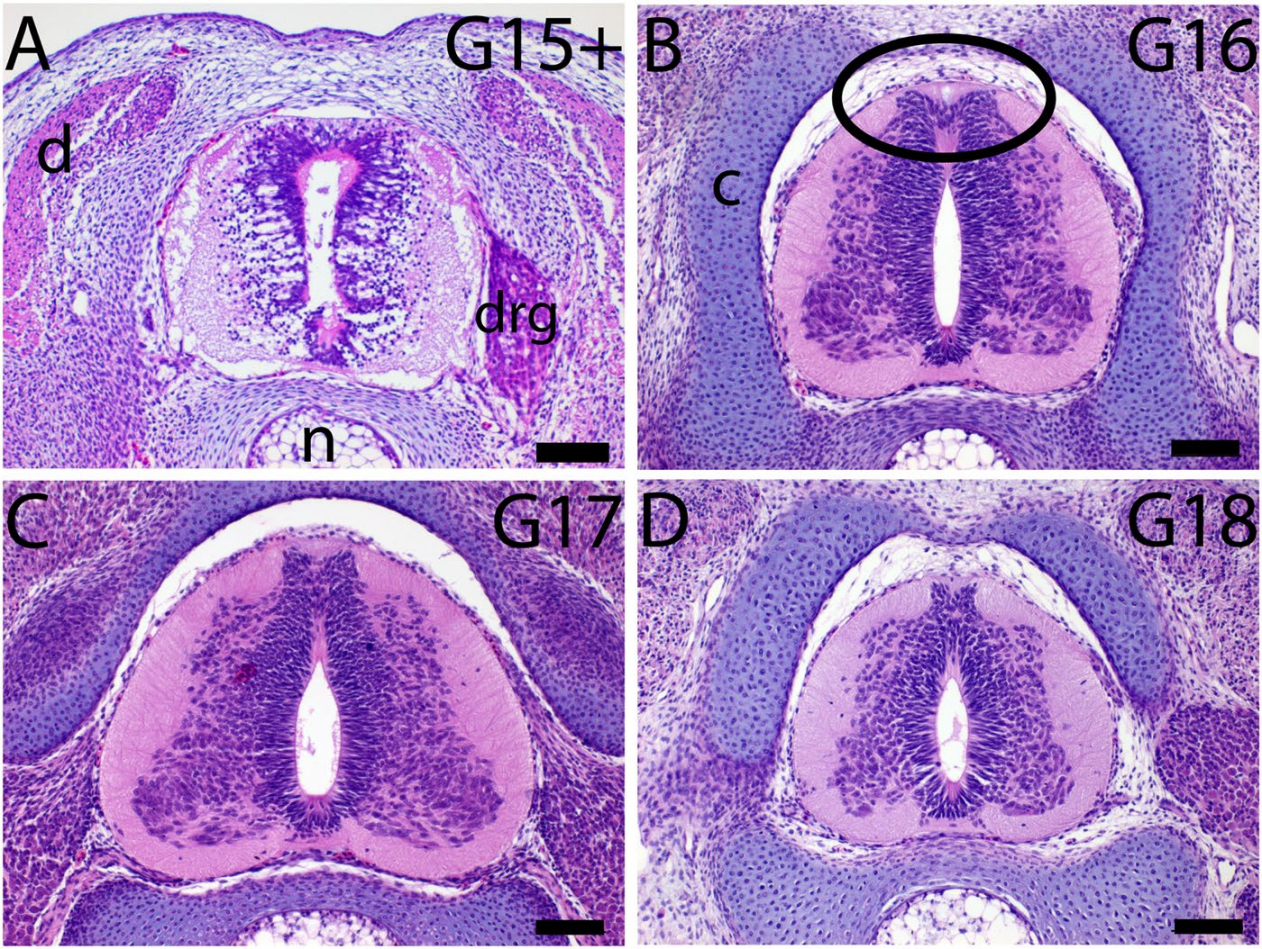
The closed neural tube developed two dorsal neuroepithelial protrusions in G16 Trachemys scripta embryo. Hematoxylin and eosin stained transverse paraffin sections demonstrated a change in the morphology of the neuroepithelial cells forming the roof plate. (**A**) At G15+, there was a uniform and rounded roof plate formed by the neuroepithelial cells. The morphology was similar to stage TK15 in the soft-shelled turtle *Pelodiscus sinensis* and stage HH28 in chick^48^. (**B**) At G16, the roof plate had two ‘folds’ of neuroepithelial cells protruding dorsally (circled). (**C**,**D**) The protruding roof plate morphology was seen also at G17 (**C**) and G18 (**D**). Scale bar approx. 100 μm (**A**–**D**). c, cartilage; drg, dorsal root ganglion; n, notochord; s, somite.

**Figure 2:**
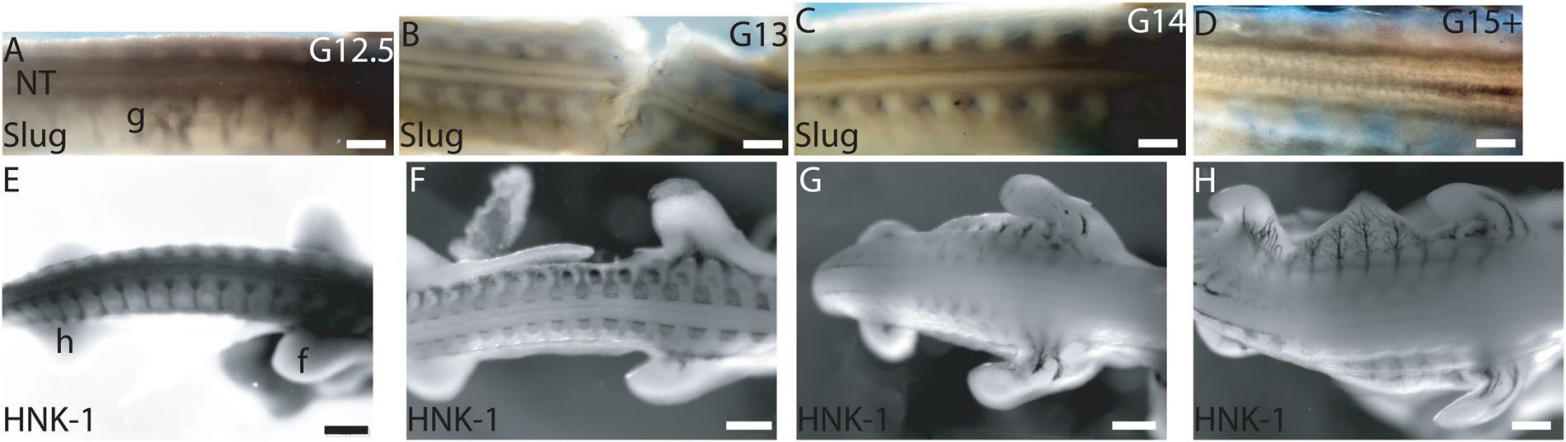
A break between the primary and secondary emigration of trunk-level neural crest cells in Trachemys scripta. (**A**) Anti-Slug antibody staining between fore- and hindlimbs demonstrated delaminating trunk neural crest cells in the dorsal neural tube at G12.5. (**B**) At G13 and (**C**) G14, Slug-positive cells were limited to neuroepithelial cells at midline of the neural tube, and no delaminating cells were visible on the dorsal neural tube. (**D**) At G15+, Slug-positive delaminating cells reappeared on the dorsal neural tube. Ganglia were Slug-positive. (**E**) Anti-HNK-1 antibody staining indicated emerging migratory cells in the dorsal neural tube at G12.5. (**F**–**H**) No HNK-1-positive migratory neural crest cells were visible in the neural tube at G13, G14 or at G15+. Ganglia and nerves were HNK-1 positive. Scale bars approx. 200 μm (**A**–**D**) and 500 μm (**F**–**H**).

At stage G15 the neural tube of *Trachemys scripta* was closed and rounded, similar to the developmental stage TK15 of a soft-shelled turtle *Pelodiscus sinensis* and a chick embryo at stage HH28^29^. A morphological change occurred in the neural tube between stages G15+ (mature G15 embryo) and G16: neuroepithelial cells lining the lumen of the neural tube had formed two dorsal protrusions at G16 that were maintained at least until G18 (Fig. 1). This morphological change coincided with the re-emergence of trunk NCC migration in turtle *Trachemys scripta* ^24,27^. Here we demonstrate that the primary NCC migration ends prior to G13 and the second wave of NCC migration commences at G15+ (Fig. 2); at G12.5 both delaminating Slug-positive^15,16^ and migrating HNK-1 positive^17–19^ neural crest cells localized to the dorsal neural tube. At G13 and G14 premigratory Slug-positive cells were confined to the midline of the neural tube suggesting that the premigratory neural crest was maintained. The second phase of delamination begun at G15+ and Slug-positive cells reappeared on the dorsal neural tube. Migratory HNK-1 positive cells reappeared on the dorsal neural tube at G16^27^. Thus, we followed the second phase trunk NCC delamination and migration from stage G15+ onwards in organ culture. We injected green fluorescent protein (GFP) expression plasmid into the lumen of the neural tube from the tail and electroporated it into one side of the neural tube. The GFP electroporation was validated on chicken embryos (SI Fig. 1). In chicken embryos, at stage HH17 migratory trunk neural crest cells are formed, and at stage HH25 no trunk neural crest cells are formed^18^; electroporation of GFP into the lumen of the neural tube at HH17 embryo *in ovo* demonstrated both delaminating and migrating cells 24 hours after electroporation that matched the migratory neural crest cells visualized by anti-HNK-1 antibody (SI Fig. 1A–D). *Ex ovo* organ culture of HH25 chicken explant, which was dissected open at ventral midline, the head and the viscera were removed, showed neuroepithelial expression of GPF 24 hours after electroporation but no cells emigrated from the neural tube (SI Fig. 1E–G).

**Figure 3:**
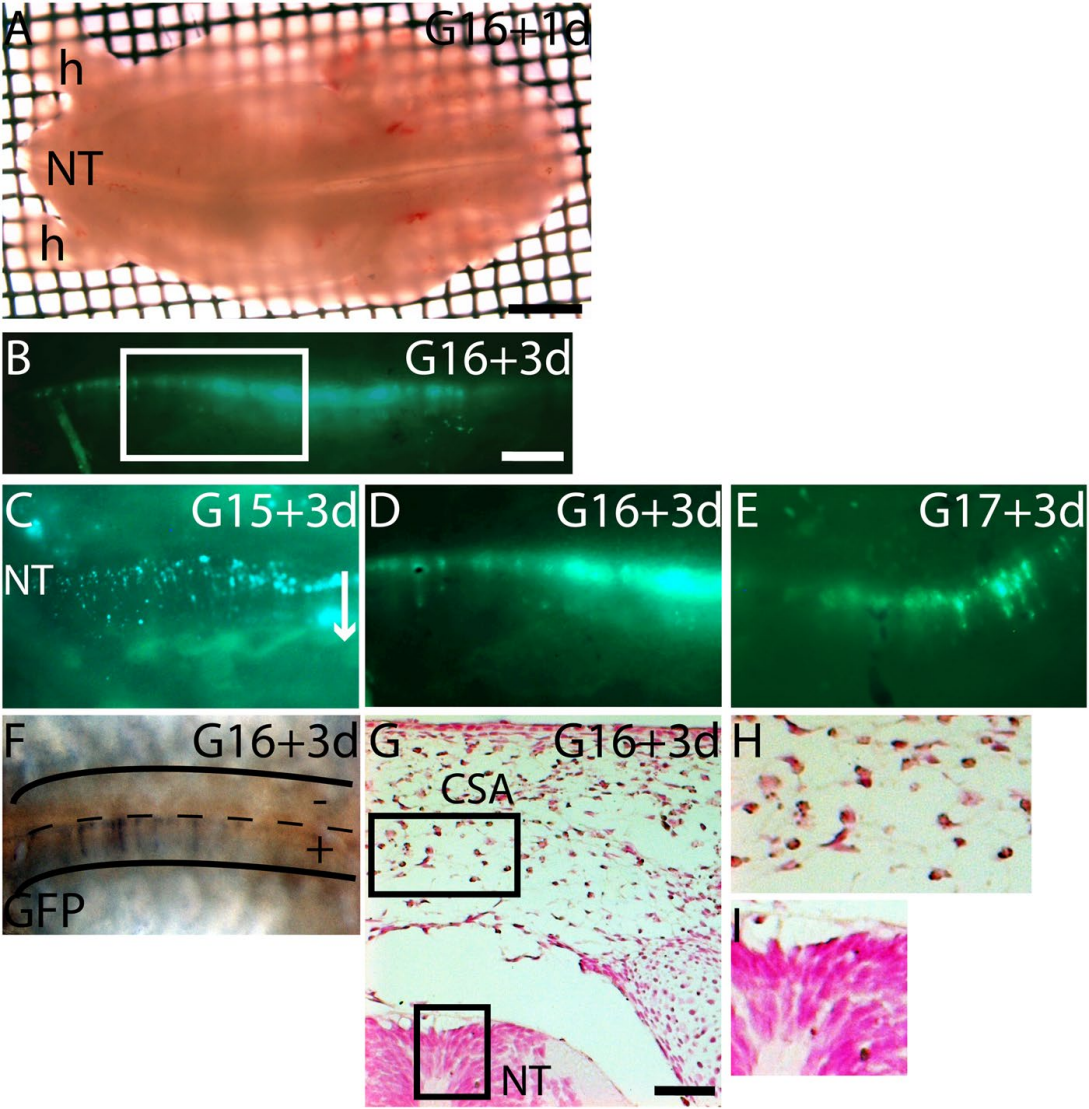
Delamination and migration of cells from the dorsal neural tube at G15-G17. GFP expression plasmid was injected into the lumen of the neural tube of turtle embryo explants and, electroporated onto one side of the neural tube; the explants were then cultured for up to three days. (**A**) Dorsal view of G16 trunk explant at 24 hpe. Hindlimbs orientate the explant: anterior to right. (**B**) G16 explant at 72 hpe demonstrating GFP expression in the neural tube along the length of the trunk-level. Boxed area is shown in higher magnification in (**D**). (**C**) G15 GFP explant at 72 hpe showing GFP-positive delaminating and migrating cells from the neural tube. An arrow shows the direction of emigration from the midline of the neural tube. (**D**) G16 GFP electroporated explant at 72 hpe showing the boxed area in (**B**). (**E**) G17 GFP electroporated explant at 72 hpe. (**F**) Bright field dorsal view of GFP-electroporated G16 trunk explant at 72 hpe stained with anti-GFP antibody. The electroporated side of the neural tube (+) showed delaminating cells along the neural tube. The control side (−) had no GFP-positive cells. (**G**) A transverse paraffin section of G16 explant at 72 hpe stained with anti-GFP antibody showing GFP-positive cells gathered at the CSA above the neural tube in the carapacial mesenchyme. Boxed area in the CSA is shown in (H) and boxed area in the NT is shown in (**I**). (**H**) Cytoplasmic expression of GFP in cells in the CSA. (**I**) Cytoplasmic expression of GFP in few cells in the dorsal neural tube. hpe, hours post electroporation; CSA, carapacial staging area for neural crest cells above neural tube in carapacial mesenchyme; NT, neural tube. Scale bar approx. 200 μm.

*Trachemys scripta* embryos were similarly dissected open at the ventral midline, and the head, the viscera, and the forelimbs were removed (Fig. 3A). The resulting explants were placed dorsal-side up onto a Trowell type organ culture, GFP expression plasmid was injected into the lumen of the neural tube through the tail and electroporated at the trunk level neural tube, the tail was removed, and the explant was cultured for up to three days. GFP-positive cells were visible on the dorsal neural tube on the electroporated side on all tested developmental stages, and the GFP expression was maintained for the duration of the culture. During culture, some fluorescent cells migrated away from the neural tube (Fig. 3B–I). Visualization of the fluorescent cells in whole mount imaging during the culture of G16 and G17 explants was limited as the surface ectoderm with forming scutes and carapacial mesenchyme, which lies on top of the neural tube, were considerably thicker than at G15 explants. Anti-GFP antibody staining confirmed the fluorescent cells in the dorsal neural fold in G16 cultured explant (Fig. 3F). GFP-positive cells were seen only on the electroporated side of the neural tube. A similar pattern of delaminating cells on the dorsal neural tube was seen in G15+ embryo stained by anti-Slug antibody: premigratory (in the midline of the neural tube) and delaminating neural crest cells (the dorsal neural tube) (Fig. 2D). The GFP-positive migratory cells grouped in the carapacial staging area (CSA) above the neural tube in the carapacial mesenchyme in G16 cultured explant (Fig. 3G,H).

Together these findings indicate that from the dorsal neural tube, a second phase trunk-level cell emigration began to emerge between G15+ and G16, and that the migratory cells accumulated into the carapacial staging area above the neural tube prior to migrating into their final destination(s).

### The second phase cell emigration from the dorsal neural tube coincides with formation of melanoblasts

We have shown neural crest cell marker Slug and HNK-1-positive cells on the dorsal neural tube and in the CSA at the time when cells were emerging from the dorsal neural tube (Fig. 2,^24,27^). No lineage analysis of the late, second phase, emigrating cells from the dorsal neural tube has been performed to confirm their neural crest nature due to technical limitations: difficulty of *in ovo* experimentation^30^; limited survival of tissue in organ culture from mid-gestation onwards; no functional genetics/transgenics available.

**Figure 4:**
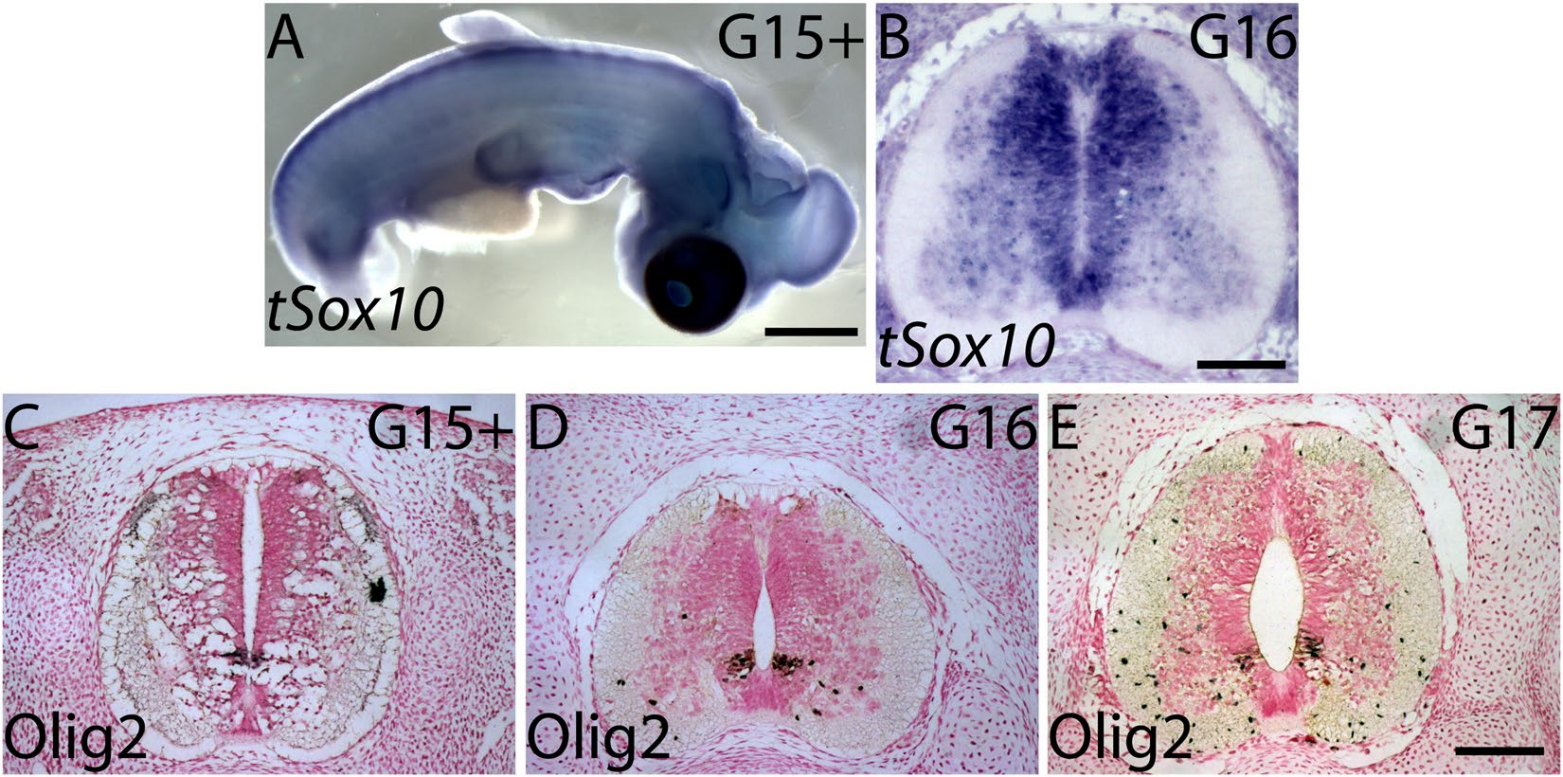
Sox10 and Olig2 expression indicated neural crest and oligodendrocyte potential at G15 and G16. (**A**) *tSox10* was expressed in the neuroepithelial cells in the neural tube at G15+. (**B**) A paraffin section of G16 neural tube showed *tSox10* expression in the dorsal and ventral neuroepithelial cells and in the migratory oligodendrocyte progenitor cells in the grey matter in the ventral neural tube. (**C**) Anti-Olig2 antibody positive oligodendrocyte progenitor cells (OPCs) in their initial domain in the ventral neuroepithelial cells at G15. (**D**) At G16, OPCs have begun to migrate ventrally. (**E**) At G17, few OPCs were also seen dorsally. Scale bars approx. 200 μm (**A**), 100 μm (**B**–**E**).

Another late developing cell type in the neural tube is the oligodendrocyte precursor cells (OPCs), which form oligodendroglia^31^. OPCs begin to develop in the ventral neuroepithelial cells, they migrate ventrally and proliferate, and their progeny migrates also into the dorsal parts of the spine^32,33^. OPCs have not been shown to express Slug or HNK-1. In both the neural crest and OPC cell lineages, Sox10 confers cell line specificity by regulating other transcription factors essential for fate-restriction: in trunk neural crest cells Sox10 regulates the expression of *Mitf*, a master switch of melanoblast differentiation, and in ventral neuroepithelial cells Sox10 is co-expressed with Olig2, a transcription factor that specifies OPCs^32,34,35^. *Sox10* was expressed both in the dorsal neuroepithelial protrusions and in the ventral neuroepithelial cells at G15+ and G16 (Fig. 4A,B). The OPC development in turtle *Trachemys scripta* was similar to other vertebrates: the initial domain of OPC formation in the ventral neuroepithelial cells expressed Olig2 at G15+ (Fig. 4C). The ventrally migrating OPCs were visible at G16 (Fig. 4D), and dorsally OPCs were seen at G17 (Fig. 4E). Therefore, the GFP-positive cells that delaminated and migrated from the dorsal neural tube (Fig. 3) did not contain oligodendrocyte precursor cells at G15+ or G16. At G17, a few of the dorsally migrating cells may have been OPCs.

**Figure 5:**
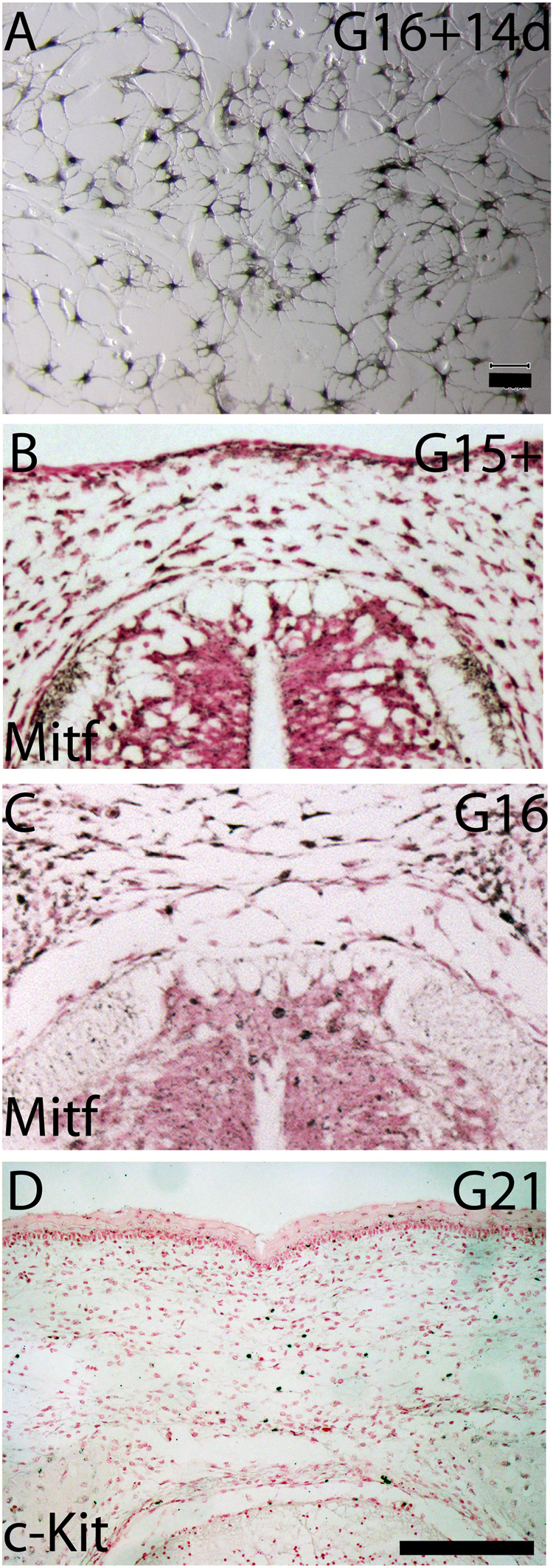
Melanoblast development coincided with the second phase cell emigration. (**A**) Pigment containing cells (black) differentiated from the migratory cells derived from G16 trunk neural tube during a 14 day cell culture. (**B**,**C**) Anti-Mitf antibody stained melanoblasts in the dorsal neural tube, the CSA, and the surface ectoderm at G15+ and G16. (**D**) c-Kit positive melanocytes were visible in the CSA above the neural tube and in the surface ectoderm at G21. Scale bars approx. 30 μm (**A**), 100 μm (**B**–**D**).

In birds and mammals, the last neural crest cells to emerge from the dorsal trunk-level neural tube are specified to become melanocytes^7,8,36^. In soft-shelled turtles, melanoblasts are seen from stage 16 onwards^10^ yet the primary trunk NCCs do not differentiate into melanocytes in cell culture unless differentiation medium is used^28^. In *Trachemys scripta*, scattered Mitf-positive cells were observed in the dorsal neural tube and in the CSA in the cultured G16 explants (SI Fig. 2). When neural tube-derived migratory cells were collected at G16 and cultured for two weeks, 8/27 samples (30%) contained pigment-producing melanocytes without exposure to specialized differentiation medium (Fig. 5A). When the migratory cells were harvested from G17 neural tubes, only 1/11 samples (9%) had pigment-producing melanocytes. Mitf-positive cells were seen in G15+ and G16 paraffin sections both in the dorsal neural tube and in the CSA (Fig. 5B,C). The relatively low numbers of melanoblasts seen in cultured G16 explants and in the cell culture samples concur with studies that have shown that a limited number of melanogenic cells are produced from the dorsal neural tube, which then expand by proliferation^36,37^. Furthermore, we have previously shown that in *Trachemys scripta*, a population of the second phase trunk neural crest cells form osteoblasts during cell culture^27^.

The G10 derived migratory cells from the neural tube, including migratory primary trunk neural crest cells, failed to differentiate into melanocytes during the two week culture. This is a result similar to stage 10 soft-shelled turtle primary trunk neural crest cells that differentiated into melanocytes in cell culture only after induction by embryo extract or melanocyte stimulating hormone^28^. In chicken embryos, trunk neural crest cells delaminate in large quantities between stages HH 15 and HH19, and melanoblasts begin to enter the dorsolateral pathway by HH17^18^. The chicken HH15 through HH19 are comparable to the soft-shelled turtle stages 9 through to advanced stage 11^29^.

These results, coupled with the earlier study by Hou and Takeuchi^28^, suggests that the trunk neural crest cells in turtles lack the endogenous potential and/or the environmental cues to differentiate into melanocytes during a similar developmental window to chick embryos, and instead melanoblast development coincides with the second phase emigration of presumptive neural crest cells from the dorsal neural tube that begins at G15+.

In chicken embryos, Sox10 expression is seen in the neural crest cells at all stages of neural crest formation, and its expression ceases in the trunk level by HH20 as the neural crest cell emigration finishes^18,38^. In turtle embryos, *Sox10* was expressed in the dorsal neural tube during the second phase emigration at G15+ and G16 (Fig. 4A,B), and Mitf-positive melanoblasts were present in the dorsal neural tube and in the CSA at G15+ and G16 (Fig. 5B,C). A melanocyte marker c-Kit is expressed in melanocytic cells in the migratory staging area in zebrafish and mice, and in melanocytic cells migrating along the dorsolateral pathway in chicken embryos^8^. In turtle embryos, few c-Kit positive cells remained in the CSA above the neural tube at G21, while c-Kit positive cells were seen adjacent to the dorsal epidermis (Fig. 5D).

**Figure 6:**
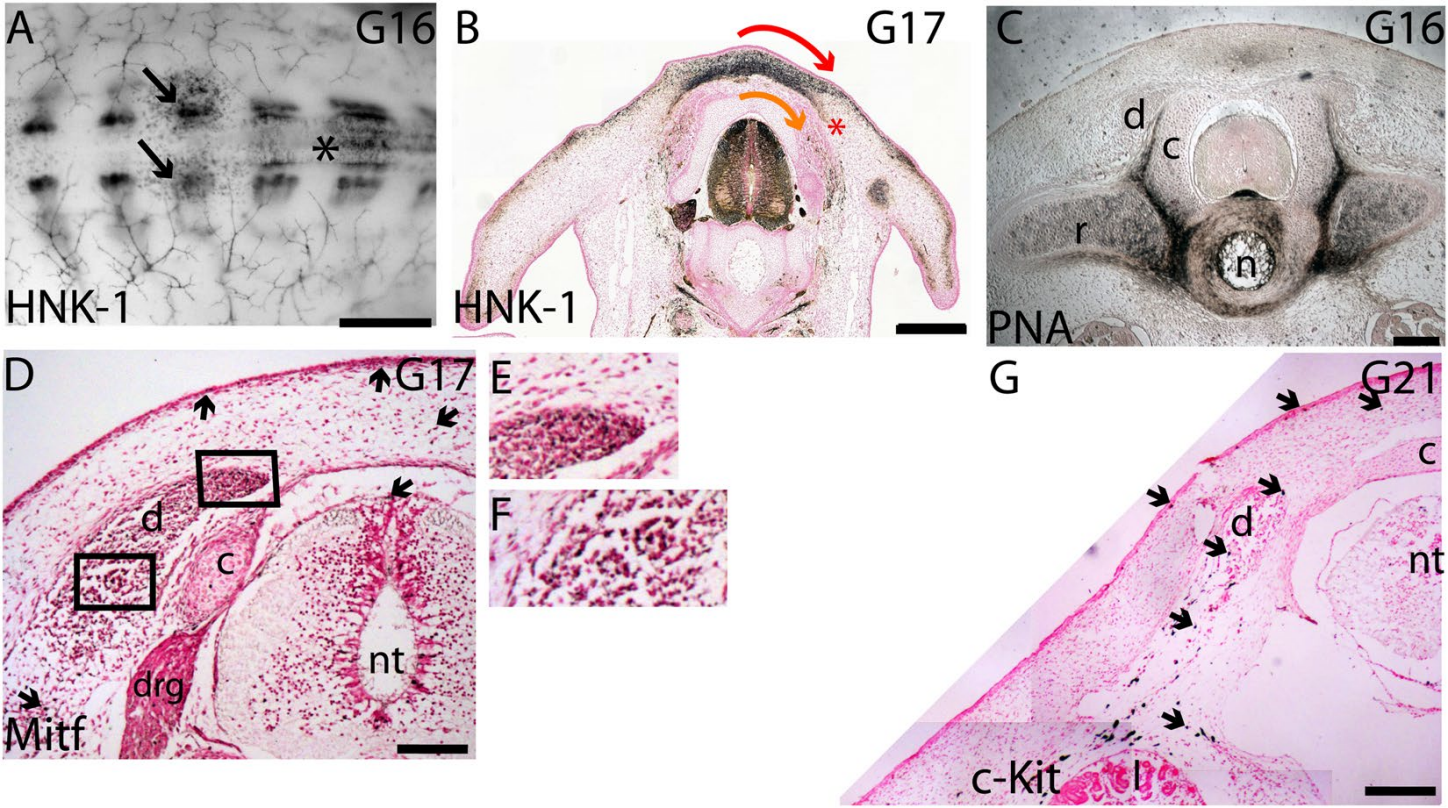
Potential migratory pathways for late neural crest cells and melanoblasts. (**A**) Anti-HNK1 antibody indicated reappearance of migratory neural crest cells in the dorsal neural tube (*) at G16. Paired round areas of HNK-1 positive cells were visible dorsolateral to the neural tube and adjacent to the surface ectoderm (black arrows). (**B**) At G17, HNK-1 positive cells appeared to follow two migratory pathways: a dorsolateral pathway underneath the surface ectoderm for some of the migratory cells and a pathway where cells travel from the CSA over the dermomyotome laterally (red arrow) or ventrally between the dermomyotome and the sclerotome (orange arrow). (**C**) PNA was not seen in the dorsal surface ectoderm, carapacial mesenchyme or in or around the dermomyotome, thus allowing entry of migratory neural crest cells to both dorsolateral and medial pathways. (**D**) Mitf-positive melanoblasts were visible on top of the neural tube, in the CSA, adjacent to the surface ectoderm and in the dermomyotome (boxed area shown in E) and in at G17. (**E**) The mediodorsal tip of dermomyotome had Mitf-positive cells. (**F**) Mitf-positive cells were detectable between the dermomyotome and the sclerotome. (**G**) At G21, c-Kit-positive melanocytes were observed in both the dorsolateral and medial pathways. Some Mitf- and c-Kit-positive cells are indicated by arrows. Scale bar approx. 1 mm (**A**,**B**), 100 μm (**C**,**D**) and 200 μm (**E**). c, vertebral cartilage; d, dermomyotome; drg, dorsal root ganglion; l, lung; nt, neural tube; r, rib.

In G16 turtle embryos, HNK-1 positive migratory NCCs were visible on the dorsal neural tube and as two round areas on both sides of the neural tube near the surface of the embryo (Fig. 6A). At G17, in a transverse paraffin section, anti-HNK-1 antibody demonstrated two possible migratory pathways for the second phase trunk NCCs: 1) a dorsolateral pathway that appeared to split into two compartments; one adjacent to the dorsal surface ectoderm and another along the ventral border of the carapace, and 2) a medial pathway between the dermomyotome and the sclerotome (Fig. 6B,^24^). In chicken and mice, neural crest-derived melanoblasts are restricted to dorsolateral pathway. The exception that confirms this generality is the Silkie strain of chicken, where melanoblasts enter ventral and medioventral regions due to an abnormal lack of PNA expression in those regions^39^. PNA-binding glycoproteins inhibit the entry of the migratory neural crest cells, especially melanoblasts, into tissues^20,39^. At G16, no PNA staining was visible in the carapacial mesenchyme or in the surface ectoderm allowing melanoblasts to enter the dorsolateral pathway. No PNA staining was visible in or around the myotome suggesting that the medial pathway was open for entry as well (Fig. 6C). The medially migrating HNK-1 positive neural crest cells could be melanoblasts on their way to internal, extracutaneous locations^10^, or they could be other neural crest-derived cells on their way to their destinations. Mitf- and c-Kit-positive cells were seen in the medial pathway at G17 and G21, respectively, indicating that some melanoblasts had entered the medial pathway (Fig. 6D–G).

**Figure 7:**
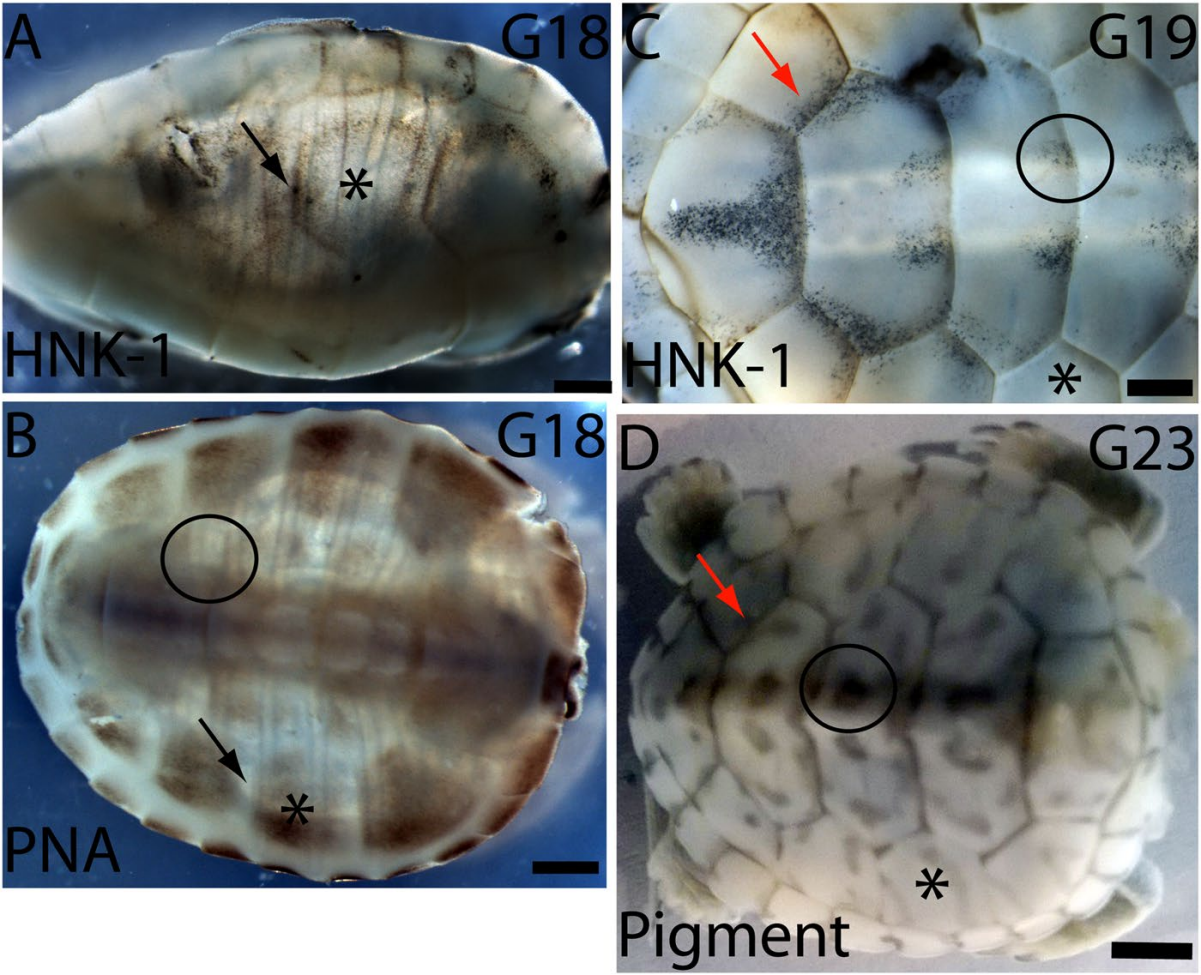
Carapacial patterning of HNK-1-positive migratory neural crest cells and pigmentation. (**A**) At G18, HNK-1 positive cells were localized to the sulci (black arrow), leaving the scutes (*) free. (**B**) The migratory neural crest cell inhibitor PNA was expressed in a mutually exclusive pattern to the HNK1-positive cells; PNA was localized to the scutes (*) leaving the sulci free (black arrow). (**C**) At G19, the carapacial HNK-1 positive cells were located in the sulci between the scutes (red arrow) and as paired spots (black circle) on both sides of the spine on the anterior end of the vertebral scutes. These areas were PNA-free in (**B**). (**D**) The pattern of pigment at G23 matched the pattern of HNK-1 at G18 and G19. Anterior is to the right in all images. Scale bars 1 mm (**A**,**D**), and 2 mm (**B**,**C**). The second costal scute on the right side of the carapace is marked by an asterisk (*) in all specimens. Black arrows indicate the sulcus between the second and the third costal scutes (**A**,**B**). Red arrows indicate the sulcus between the fourth costal and the fourth vertebral scutes (**C**,**D**). The black circle shows one of the paired HNK1-positive migratory neural crest cell spots on the fourth vertebral scute in (**C**), and the same location of pigment in (**D**).

A correlation was observed between the carapacial location of the HNK-1-positive migratory neural crest cells on the dorsolateral pathway and the epidermis-associated pigment cells appearing later in development (Fig. 7). At G18, migratory HNK-1 positive neural crest cells were limited to the periphery of forming scutes and to the sulci between the scutes (Fig. 7A) while PNA-binding proteins were expressed on most of the scute epithelium (Fig. 7B). HNK-1 positive cells continued to be located in the sulci and in parallel patches on both sides of the neural tube at G19 (Fig. 7C,^24^). The pattern of HNK-1 positive cells at G18 and G19 matched the pattern of pigment (formed by the melanocytes) that was developed by G23 (Fig. 7D). The pattern of HNK-1 positive cells and pigment was mutually exclusive to that of PNA staining, implying that PNA-binding proteins may have been providing an inhibitory barrier that directed the patterning of neural crest-derived melanocytes on the carapace.

## Discussion

Melanocytes produce the pigment melanin, and in turtles melanin is found throughout the integument – in the skin and in membranes covering organs^10^. Melanin and its patterning are important in the animal’s adaptation to its environment and social contacts. In cold-blooded animals, melanin also plays an important role in thermoregulation^1^. Here we studied the formation of melanocytes in the cold-blooded turtle *Trachemys scripta*.

At the time of migration, neural crest cells form a heterogeneous cell population that includes cells that are multi- or bipotent and cells that are fully committed. Trunk neural crest cells differentiate into neurons, glial cells of the peripheral nervous system, skin melanocytes and adrenergic cells during different times of development^40,41^. Some of the multipotent premigratory neural crest cells become fate-restricted to a melanoblast/glial bipotent progenitor cells, and some of these bipotent cells will be specified as melanoblasts. The fate specification of melanoblasts occurs prior to delamination from the neural crest; melanoblasts accumulate into the migratory staging area, and migrate along a dorsolateral pathway and differentiate into melanocytes^36^. Turtle embryos have two distinct windows of trunk neural crest cells emigration^24,27,42^. The first window, around G12, resembles those of chicks and mice and contains the neural precursors, and the second window, beginning at mature G15, represents a turtle-specific emigration of neural crest cells (Fig. 2;^27,42^). The second phase of trunk neural crest cell emigration occurs after the sensory and enteric neurons have formed. Previously, we have demonstrated that a population of these cells are capable of forming osteoblastic cells during cell culture^27^. Here, we show that the precursors of the turtle melanocytes developed during the second window of trunk neural crest emigration. Melanogenic Mitf-positive cells emigrated from the dorsal neural tube at G16 (Fig. 5, SI Fig. 2). Once trunk neural crest cells segregate from the neural tube, they become at first more rounded and irregular in shape with increasing number of filopodia protruding out of the neural tube^43^. Here we show a morphological change in the dorsal neuroepithelial cells in the spinal cord and rounded cells with long filopodia in the CSA above the spinal cord (for instance Figs 1, 3, 4). In organ culture, we were able to follow cells leaving the dorsal neural tube and enter the carapacial staging area at stages G15+ to G17. In *Trachemys scripta* embryos, Mitf-positive melanoblasts were present in the CSA at G15+ and G16, and pigmentation was seen from G19 onwards (Figs 4–5). Some mature c-Kit-positive melanocytes remained in the CSA at G21 suggesting that few cells had yet to enter their migratory pathway (Fig. 5D).

In avian embryos, the trunk neural crest cells that are restricted to neuronal and pigment cells are produced at the end of the migratory phase and move along the dorsolateral pathway^41^. Here we show that migratory cells harvested from the trunk-level neural tube at G16 contained a subpopulation of cells that readily differentiated into melanocytes during a 14-day cell culture experiment (Fig. 5). In addition, Mitf-and c-Kit-positive melanocytic trunk neural crest cells were detected on both dorsolateral and medial pathways (Fig. 6). The medial pathway may be responsible for the extracutaneous melanocytes found in soft-shelled turtles^10^. In chicken embryos, a PNA-dependent migratory staging area for presumptive melanoblasts has been observed^9,20^. Turtles appear to have extended the timing of melanoblast release, and an extensive carapacial staging area has been formed above the neural tube, and this area was devoid of inhibitory PNA-binding glycoproteins (Fig. 6).

Turtles appear to differ from birds and mammals in that their melanocytic neural crest cells are allowed to enter not just the dorsolateral migration pathway but also the medial pathway (Fig. 6,^10^). In the mutant Silkie chicken embryos, melanoblasts develop normally and their initial dispersal from the neural tube follows the same migratory pathways as in wild type chicken and quail embryos^39,44^. A difference in the migration pathways between the mutant Silkie and other wild type chicken embryos was noted from stage HH22 onwards: neural crest cells continue their migration medioventrally in the Silkie embryos and end up in internal locations, including the sclerotome, dorsal aorta and kidney. This continued medioventral migration of neural crest cells correlates with the lack of a PNA barrier dorsally and laterally around the neural tube in the Silkie embryos. In wild type chicken embryos, PNA-binding proteins localize in a dorsal and lateral band round the neural tube extending to the perinotochordal mesenchyme at HH28^44^. Here we have shown that in G16 turtle embryos, as in Silkie embryos, PNA-binding proteins were not expressed by the surface ectoderm, the carapacial mesenchyme, the dermomyotome or the sclerotome, suggesting that all neural crest migratory pathways were accessible to melanoblasts (Fig. 6C). The pattern of HNK-1 positive migratory neural crest cells, which matched the pattern of pigment later in development, was mutually exclusive to the pattern of PNA-binding proteins (compare Fig. 7A and B).

Although the turtle is an interesting model animal and has its strengths in helping us to understand the evolutionary developmental biology, it has its limitations as an experimental animal model; difficulty of *in ovo* manipulation and culture, difficulty of organ cultures past mid-gestation due to the size of the tissue, and the absence of transgenic animals^30^. Thus, we have not been able to provide cell lineage analysis of the second phase emigrating cells to validate whether these cells truly are neural crest cells and what derivatives they provide in the developing turtle. However, we have looked at morphology, used molecular markers, cultured cells and organs, and put the findings in context with what is known in other vertebrates. For instance Mitf-positive cells in the dorsal neural tube have been shown to be of trunk neural crest origin in mice, chicken, fish and frogs^7,36^. Here we have shown cells emigrating from the dorsal neural tube at the developmental stages when Mitf-positive cells appeared in the dorsal neural tube in fixed embryos and in organ cultured explants. Also, a subpopulation of migratory cells collected from the trunk-level neural tube differentiated into pigment cells in cell culture without prompting. The appearance of Mitf-positive cells in the dorsal neural tube and in the above-lying carapacial mesenchyme overlapped the expression of *Sox10*, a transcription factor needed to maintain neural crest^13^ and to switch on the expression of Mitf in melanoblast precursor cells that are derived from the neural crest^35^. Mitf expression also coincided with Slug- and HNK-1-positive cells in both locations. Slug and HNK-1 have been used to demonstrate the presence of premigratory and migratory trunk neural crest cells, respectively, in several vertebrates including turtles^15,17–19,42,45^.

Thus, we feel confident that the dorsal neural tube-derived melanoblasts in turtles are similar to those in other vertebrates: they are derived from a subset of presumptive neural crest cells that have been fate-restricted to melanoblasts, pause in a staging area, and enter the migration pathways open for them. Turtles are set apart from other amniotes (with the exception of the Japanese Silkie chicken) by (1) the expression pattern of inhibitory PNA-binding proteins, and (2) the incorporation of the melanoblast development into the later (turtle-specific) stage of cell emigration from the trunk dorsal neural tube.

## Methods

### Material

*Trachemys scripta elegans* eggs were purchased from the Kliebert Turtle and Alligator Farm (Hammond, LA, USA). Animal work was carried out in accordance with the guidelines and approval from Finnish National Board of Animal Experimentation. Eggs were incubated in a humidified incubator at 30 °C, and embryos were staged according to Greenbaum (G)^46^. Total RNA was isolated from developmental stage G14 and G17 embryos and reverse transcribed into cDNAs that were used as templates to clone *T. scripta* specific *Sox10 (tSox10)*. Primers were designed against sequences in *T. scripta* transcriptome^47^. PCR products were purified and ligated into a vector, and resulting plasmids were sequenced. DIG-labeled antisense cRNA probes were transcribed from linearized plasmid, and *in situ* hybridization on 4% PFA (wt/vol)-fixed paraffin sections or whole mount samples were performed according to standard protocols.

### Organ culture and GFP-transfection by electroporation

*Trachemys scripta* embryos were rinsed and dissected in PBS with anti-fungal agents. Only viable embryos (*i.e*. heart beating) were used. Dissected trunk explants were placed dorsal side up on nucleopore filters supported by grids in Trowell type organ culture system. Hindlimbs were left on explants for orientation. Culture medium (DMEM, 10% (wt/vol) FCS, GlutaMAX-1, 100 μg/ml ascorbic acid, 20 IU/ml penicillin-streptomycin, and anti-fungal reagents) was changed daily. Explants were cultured at 30 °C in 5% CO_2_. GFP plasmid (400 ng/μl in PBS with Fast Green Dye) was injected into the lumen of the neural tube from the posterior end of the neural tube (the tail) with a microcapillary pipette to fill the lumen throughout the neural tube. The surface of the explant was rinsed with PBS prior to electroporation to wash off any trace amounts of GFP plasmid and tail was removed. The electrodes were placed dorsally on both sides of the neural tube to ensure the direction of GFP plasmid-intake to be only on one side of the neural tube at the site of electroporation by BTX ElectroSquare porator ECM830, Mode LV: electric pulses (7 pulses, 50.6 ms at 629.7 ms interval) at 18–22 V were given depending on the developmental age of the explant.

### *In vitro* cell culture of trunk-level migratory neural tube cells

Trunk-level neural tubes were dissected and cultured until cells migrated onto fibronectin- and poly-D-lysine-coated culture dishes as previously described^27^. Briefly, neural tubes were removed from the culture dishes after 2–3 days of culture, and the remaining cells were cultured for 14 days in the presence of 10 ng/ml FGF2. Melanocytes were identified due to the presence of black pigment.

### Immunohistochemical staining

4% PFA-fixed paraffin sections were dewaxed, rehydrated, treated to block endogenous peroxidase activity, boiled in citrate buffer pH 6.0 for antigen retrieval (except for PNA staining), preblocked with 0.3% BSA, and incubated with anti-c-Kit (1:500, Abcam cat# ab 32363), anti-GFP (1:1000, Chemicon cat# MAB3580), anti-HNK1 (1:1000, BD Biosciences cat# 559048), anti-Mitf (1:500, Bioss cat# bs-1990R), PNA (peanut agglutinin) (1:500, Sigma cat# L7759), or anti-Slug (1:500, CST cat# 9585) antibody overnight at 4 °C, washed and incubated with the appropriate secondary HRP-conjugated antibody (1:1000) overnight at 4 °C. Antibody detection was performed by enzyme metallography with manufacturer’s protocol (EnzMet kit, Nanoprobes) and the sections were counterstained with nuclear fast red or hematoxylin.

### Data availability

Materials and protocols available upon request from the corresponding author.

## Electronic supplementary material

Supplementary information
