# Supplementary material for: Melanoblast development coincides with the late emerging cells from the dorsal neural tube in turtle Trachemys scripta
Source: Sci Rep. 2017 Sep 21;7:12063. doi: 10.1038/s41598-017-12352-0 (PMC5608706; doi:10.1038/s41598-017-12352-0)

Melanoblast development coincides with the late emerging cells from the dorsal neural tube in turtle *Trachemys scripta*

Ritva Rice[1,2*], Judith Cebra-Thomas[3], Maarja Haugas[4,#], Juha Partanen[4], David P. C. Rice[2,5], Scott F. Gilbert[1,6]

[1]Developmental Biology, Institute of Biotechnology, University of Helsinki, Finland

[2]Orthodontics, Department of Oral and Maxillofacial Diseases, University of Helsinki, Finland

[3]Department of Biology, Millersville University, PA, USA

[4]Department of Genetics, University of Helsinki, Finland

[5]Orthodontics, Oral and Maxillofacial Diseases, Helsinki University Hospital, Finland

[6]Department of Biology, Swarthmore College, PA, USA

[*]Correspondence to ritva.rice@helsinki.fi

[#]Present address: Institute of Biomedicine and Translational Medicine, University of Tartu, Estonia

Supplemental information (SI) figure legends.

SI Figure 1. Electroporation of GFP expression plasmid into the lumen of chicken neural tube and cultured for 24 hours. (A, B) Stage HH17 chicken embryo electroporated and cultured *in ovo* had delaminating and migratory trunk neural crest cells. (C) Anti-GFP antibody staining of the same specimen shown in (A) confirmed the delamination of the trunk neural crest cells along the dorsal neural tube on the electroporated side and the segmental migration of the neural crest cells. (D-F) HH25 chicken explant was electroporated and cultured *ex ovo* for 24 hours in a Trowell type organ culture. GFP expression was visible in the neuroepithelial cells in the middle of the neural tube and no delaminating or migrating cells were seen. (G) Stage HH17 fixed chicken embryo stained with anti-HNK-1 antibody. Pattern of migratory neural crest cells matched the GFP electroporated sample shown in (A-C). Scale bars 500 μm.

SI Figure 2. Potential cell differentiation in G16 carapacial explants cultured for three days. (A) Few Mitf-positive cells (black) were seen in the CSA (boxed area shown in B) and in the dorsal neural tube (boxed area shown in C). Mitf-positive cells were also seen in the surface ectoderm and in the dermomyotome. (D) HNK-1 positive migratory neural crest cells (white) shown in a dark field image. C, vertebral cartilage; CSA, carapacial staging area; NT, neural tube; S, somite. Counterstain Nuclear Fast Red in (A).

SI_Rice_Figure1

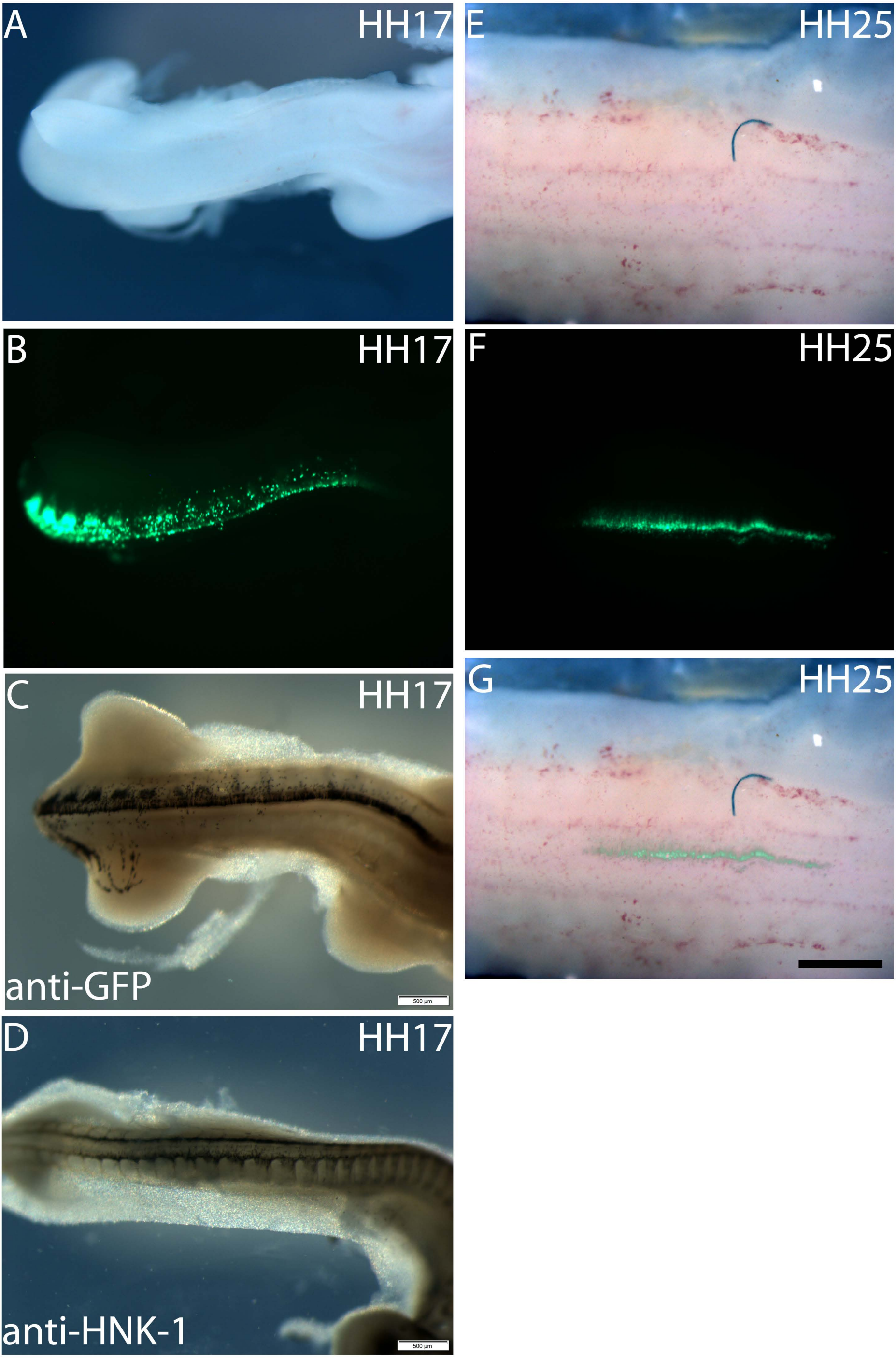

SI_Rice_Figure2

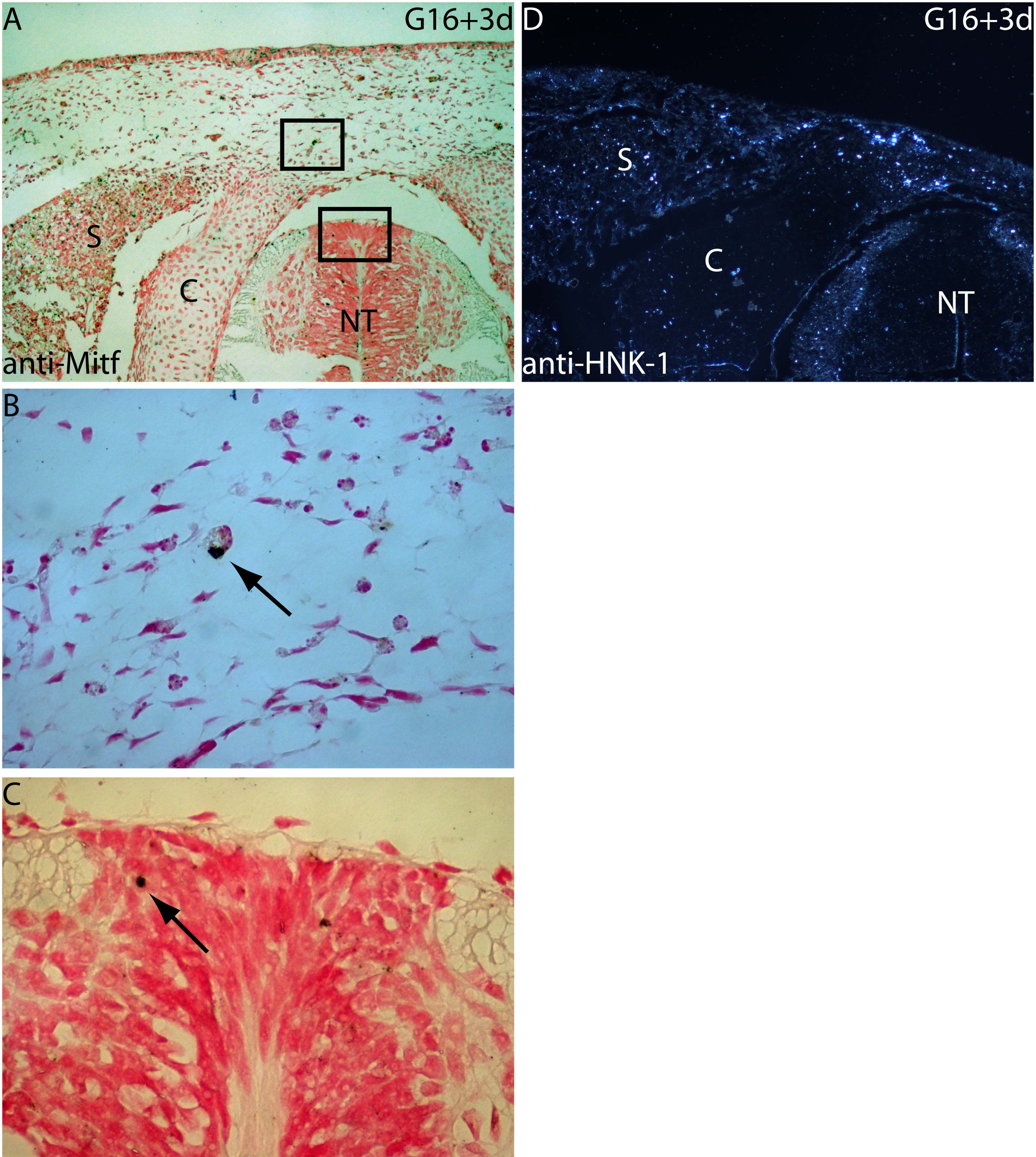

Supplement: Supplementary file 1 — Supplementary information [file 41598_2017_12352_MOESM1_ESM.pdf]
